# A Study of the Relationships between Serum Calcitonin Gene-Related Peptide, Sex Hormone, Homocysteine and Coronary Heart Disease in Postmenopausal Women

**Published:** 2006-02

**Authors:** Wang Zhen, Guo Jingxuan, Wang Xian, Zhao Yiming, Hou Lingfei

**Affiliations:** *Department of Emergency, Peking University Third Hospital, Beijing, P. R. of China*

**Keywords:** coronary heart disease, calcitonin, gene-related peptide, homocysteine, estradiol postmenopausal women

## Abstract

**Objective:**

To investigate the relationships among serum calcitonin gene-related peptide (CGRP), homocysteine (Hcy), female hormone, and coronary arteriopathy in postmenopausal women.

**Method:**

In a cross-sectional study, serum CGRP, estrodiol (E2), progesterone (P) and Hcy levels of 144 postmenopausal women with coronary heart disease (CHD) and non-coronary heart disease (NCHD) were measured.

**Results:**

The mean serum CGRP level was significantly lower in CHD patients than in NCHD subjects. The mean serum E2 and P level were significantly lower in CHD patients than in NCHD subjects. The mean serum Hcy level was significantly higher in CHD patients than in NCHD subjects. By multivariate logistic regression, the OR of high Hcy level ≥1, *p*<0.01, which suggests that Hcy is an independent risk factor in the development of coronary arteriopathy. The OR of CGRP, E2, and P are all≤1, indicating that CGRP, E2 and P are independent protective factor.

**Conclusion:**

The results of our study show that Hcy is an independent risk factor in the development of arteriopathy. CGRP and endogenous E2 are independent protective factors in the development of coronary arteriopathy. There are no relationships between Hcy, CGRP, and endogenous E2.

## INTRODUCTION

Numerous possible risk factors for coronary heart disease (CHD) have been identified, of which include smoking, physical inactivity, gender, obesity, age, hypertension, hyperlipemia and diabetes. In the 1990s many reports associated elevated serum or plasma homocysteine (Hcy) levels with CHD, suggesting that Hcy is a potent inducer of atherosclerosis (1-4). The results of our previous studies also confirm theses findings (5). Calcitonin gene-related peptide (CGRP) is a 37-amino acid neuropeptide mainly present in sensory nerve fibers, which is present in almost all mammalian organs, including humans. It is widely known that CGRP has various protective effects on the cardiovascular system (6-7).

Hormonal decline is an endocrine characteristic of postmenopausal women, who show a general decrease in female gonad hormones. Recent studies have found that the decline of gonad hormone levels with age is accompanied by an increase risk of cardiovascular diseases. Furthermore, some reports have shown that hormone replacement therapy reduces Hcy levels (8-9), while other studies have shown no correlation between hormone replacement therapy and Hcy (10-11) in postmenopausal women. Some reports have demonstrated that E2 affects CGRP level in animals (12-13).

Whether the decreases in endogenous female gonadal hormones are involved in the age-related alterations of Hcy and CGRP remain unknown. There has also been little reported on the relationships between serum Hcy, serum CGRP, and endogenous female gonad hormone levels. The present study is designed to determine the relationships among serum Hcy, serum CGRP, endogenous female gonad hormone, and coronary arteriopathy in postmenopausal women.

## METHODS

### Case ascertainment and control identification

One hundred and forty four postmenopausal women from a consecutive series of subjects were enrolled in the study. The ages of the postmenopausal women ranged from 51-73 years, with absence of menstrual bleedings for at least one year. They were all with chest pain that suggest CHD or myocardial infarction (MI) undergoing diagnostic coronary angiography for investigation of chest pain. CHD indicates those subjects, the ages of postmenopausal women ranged from 56-72 years, with a mean ± standard deviation (SD) age of 63.9 ± 6.5 years, whose scores for severity of coronary arteriopathy≥1. NCHD indicates those subjects, the ages of the postmenopausal women ranged from 50-71 years, with a mean ± standard deviation (SD) age of 58.9 ± 5.4 years, whose scores for severity of coronary arteriopathy=0. All two groups had no history of renal, hepatic, endocrinologic, gastrointestinal disease. They all did not take any estrogen and progestogen therapy. All patients were evaluated for CHD risk factors. Clinical details, including conventional risk factors for CHD (age, smoking, drinking, previous MI, family history for premature CHD, hypertension, diabetes, hyperlipemia, drug therapy, blood pressure, height and weight) were recorded for all patients by interviews. Diagnostic coronary arteriography was carried out in all CHD and NCHD patients. The World Health Organization criteria verified definite or possible MI based on chest pain symptoms, electrocardiogram changes, and enzyme determination were used to define previous MI (14). Body mass index was calculated (kg/m2).

### Interpretation and scoring of coronary angiograms

Angiographic scoring for each vessel segment was based on the severity and extent of disease observed in the “most severe” projection. For the purpose of analysis, the presence of angiographically detectable CHD was defined by an extent score. All angiograms were reviewed by two experienced cardiologists who had no knowledge of the patients clinical history and laboratory results. Scoring of severity of coronary artery disease was performed with a modification of the coronary atherosclerosis scoring system described previously (15). The percentage by which each lesion in the proximal coronary circulation narrowed the artery was assessed according to the maximal narrowing of the diameter of the artery in all projections. The extent and severity of the proximal coronary disease was assessed by assigning points to each lesion as follows: less than 50% stenosis of the luminal diameter, 1 point; 50% to 74% stenosis, 2 points; 75% to 99% stenosis, 3 points; total obstruction, 4 points. The points for each lesion in the proximal coronary circulation were summed and a score for severity of coronary atherosclerosis was obtained.

### Laboratory measurements

All venous blood specimens were drawn from patients at 8:00 a.m. after 12 hours fast before angiography. Blood samples were centrifuged in low temperature within one hour. Part of the serum was used to determine serum lipids, and the remaining was stored at -30C for subsequent analysis for Hcy, estradiol (E2), progesterone (P), and CGRP. Serum lipids were measured with Technicin Autoanalyzer 7010A (Hitachi, Japan). High-density lipoprotein cholesterol (HDL-C), low-density lipoprotein cholesterol (LDL-C), total cholesterol (TC) and triglyceride (TG) were measured by enzymatic method.

Hcy were measured using high–performance liquid chromatography (HPLC) with 420-AC fluorescence detection (Waters, U.S.A) and HPLC reagent kits (Bio-Rad, U.S.A).

The radioimmunoassay (RIA) technique used for the measurement of CGRP levels in the present study was similar to our previous reports (16). Briefly, standards of synthetic CGRP (rat amino acid sequence, Peninsula Laboratory, USA) ranging from 2.5 to 1000 pg/assay tube or the dried samples were reconstituted in a volume of 200 μl RIA buffer (0.1 M, pH7.4, phosphate buffer containing 0.1% BSA, 0.01% NaN3, 50 mM NaCl, 0.1% Triton X-100) and incubated for 24 h at 4°C with 100 μl anti-CGRP antibody (Peninsula Laboratory, Belmont, CA, USA) diluted in RIA buffer. The mixture was then incubated for another 24 h at 4°C with 100 μl 125I-labelled CGRP (125ICGRP (human sequence) was purchased from Amersham (Arlington Heights, IL, USA).) in RIA buffer. Free and bound fractions were separated by adding 100 μl goat anti-rabbit IgG and normal rabbit serum incubate at room temperature for 2 h. An additional 0.5 ml RIA buffer was added, and the RIA test tubes were centrifuged (2800× g, 4°C) for 20 min. After removal of the supernatant, the RIA test tubes were counted by (gamma)-counter. The IC50 values for the CGRP RIA were 30–40 pg/RIA tube. The intraassay coefficient of variation was 5.1%. For the intraassay coefficients of variation, the corresponding values 4.5%.

Concentration of E2 and P were measured by using Immulite Automated Immunoassay System (DPC Inc. American). The intra-assay and inter-assay variability was less than 5%.

Statistical analyses. Statistical analysis was performed with SPSS 10.0 (Statistical computer package for the Social Science; SPSS Inc., Chicago, IL.) Data of continuous variables are expressed as mean (± standard deviation (SD)). Data were analyzed by one-way analysis of variance (ANOVA). Student’t test for means between two groups. *P*<0.05 indicates significant difference. Data were further analyzed by logistic regression for multiple comparisons. A logistic analysis remains an effective statistical tool for investigating the role of a series of measured independent variables. That is, the relationship among variables is modeled by an additive logistic equation. Then coefficient of a sequential analysis is given with forward method to identify if the factor is independent or not. In logistic regression model, if OR of a variable>1, means this factor is a risk factor, otherwise, OR<1, a protect factor (17).

## RESULTS

Descriptive characteristics of subjects in the study are listed in Table 1. Subjects of CHD group have high rates of suffering diabetes and hypertension, there were significant difference compared with NCHD group. *P*<0.05. Ages of CHD group were higher than NCHD group, *P*<0.05. Subjects of CHD group have higher serum Hcy level than NCHD group. Serum CGRP level, E2 level and *P* level in two groups showed an opposite trend compared with Hcy level. CHD group have lower serum CGRP level, E2 level and *P* level than NCHD group. Serum TG level was higher in CHD group compared with NCHD group, *P*<0.05. There were no difference in serum TCHO, LDL-C, HDL-C and BMI between CHD and NCHD group.

**Table 1:** Comparison of Clinical characteristics, lipoprotein profile, Hcy, CGRP, E_2_ and P levels (x ± SD)

Parameter	CHD (n=75)	NCHD (n=69)	*P* Value

Age (years)	63.9 ± 6.5 b	58.9 ± 5.4	<0.05
BMI (Kg/m^2^)	24.1 ± 3.7	25.3 ± 4.8	>0.05
No. of Hypertension (%)	33% (25/75) b	13% (9/69)	<0.05
No. of Diabetes (%)	24% (18/75) b	8.7% (6/69)	<0.05
TCHO (mmol/l)	5.54 ± 1.98	5.13 ± 1.12	>0.05
TG (mmol/l)	2.29 ± 1.84 b	1.79 ± 0.98	<0.05
LDL-C (mmol/l)	2.99 ± 1.02	2.95 ± 0.83	>0.05
HDL-C (mmol/l)	1.19 ± 0.4	1.16 ± 0.36	>0.05
Hcy (umol/l)	15.3 ± 6.5 b	10.2 ± 2.8	<0.05
CGRP (ng/L)	103.6 ± 59.8 a	164.6 ± 50.7	<0.01
E_2_(pmol/L)	67.9 ± 24.4 a	91.7 ± 23	<0.01
P (nmol/L)	0.89 ± 0.46 b	1.11 ± 0.45	<0.05

Values are means ± SD. CHD, Postmenopausal women with CHD; NCHD, Postmenopausal women without CHD.

a Compared with NCHD, *P*<0.01;

b Compared with NCHD, *P*<0.05.

Single factor analysis showed elevated Hcy level, higher ages, hypertension, diabetes, lower CGRP level, E2 level and *P* level having relationship with CHD. That means all these factors may have influence on the generation of arteriopathy. But which one is independent risk factor? Which one is not? These factors contribute unequally to the outcome (CHD). Therefore, single factor analysis could not answer this question. An extremely useful approach to identifying the relationship among multivariate measurements involves the application of logistic regression model. It gives a comprehensive picture of the application of logistic model to explore the relationships with multivariable data. In order to understand the effects of elevated Hcy level and other factors, the data were further analyzed by multivariate logistic regression model (Table 2). We can found that when Hcy alone entering the regression model, the OR of Hcy≥1, the difference is very small, *p*<0.01. We can identify that elevated Hcy level was an independent risk factor in the development of women CHD. Similarly, when old age, hypertension, diabetes, TG alone entering the regression model, the OR of old age, hypertension, diabetes, TG were all ≥1, the difference is very small, *p*<0.01. We can identify that old age, hypertension, diabetes, TG were all independent risk factor in the development of women CHD. This is similar with the results of single factor analysis. Oppositely, when CGRP alone entering the regression model, the OR of CGRP ≤1, the difference is very small, *p*<0.01. In accordance with CGRP, when E2 and P alone entering the regression model, the OR of CGRP ≤1, the difference is very small, *p*<0.01. We can identify that CGRP, E2 and P were all independent protective factor in the development of women CHD. This is similar with the results of single factor analysis also.

**Table 2:** Variables and contents in logistic regression model

Variables	Contents

Age (years)	×1 Continuous (years)
BMI	×2 Continuous (Kg/m^2^)
Hypertension	×3 Binary (0=No 1=Yes)
Diabetes	×4 Binary (0=No 1=Yes)
TCHO	×5 Continuous (mmol/l)
TG	×6 Continuous (mmol/l)
LDL-C	×7 Continuous (mmol/l)
HDL-C	×8 Continuous (mmol/l)
Hcy	×9 Continuous (umol/l)
CGRP	×10 Continuous (ng/L)
E_2_	×11 Continuous (pmol/L)
P	×12 Continuous (nmol/L)

This study aimed to find the relationship among Hcy, CGRP, and E2. Therefore with forward method, from Table 3-5, we can found that when hypertension entering regression model, the OR of hypertension ≥1, the difference is very small, *p*<0.01. But when CGRP entering the regression model, the OR of hypertension changed greatly, *P*>0.05, showing hypertension has the similar effects compared with lower CGRP level. That means lower CGRP level and hypertension were all independent risk factor in the development of women CHD. Similarly, when age, TG and diabetes entering the regression model, the OR of TG, age and diabetes were all≥1, *p*<0.01. But when CGRP entering the regression model, the OR of age, TG and diabetes changed greatly, *P*>0.05. That means lower CGRP level, TG, high age and diabetes were all independent risk factor in the development of women CHD. Above analysis showed that age, diabetes, hypertension, TG and lower CGRP level have similar effects.

**Table 3:** Variables in the equation

Variables	Coefficient	SE	P Value	Odds Ratio	95% CI for Coefficient	
					Lower	Upper

Hcy	0.264	0.068	<0.001	1.302	1.140	
Constant	-3.679	0.837	<0.001	0.025		

**Table 4:** Variables in the equation

Variables	Coefficient	SE	P Value	Odds Ratio	95% CI for Coefficient	
					Lower	Upper

E_2_	-0.052	0.012	<0.001	0.949	0.928	0.971
Constant	4.643	1.132	<0.001	103.83		

**Table 5:** Variables in the equation

Variables	Coefficient	SE	P Value	Odds Ratio	95% CI for Coefficient	
					Lower	Upper

CGRP	-0.022	0.006	<0.001	0.978	0.967	0.989
Constant	2.501	0.765	<0.001	12.19		

To study the relationship among CGRP, Hcy and female gonad hormone, with forward method, they were also analyzed by logistic regression model (Table 6). The last main logistic regression model involves CGRP, Hcy and E2. We can find that when CGRP and Hcy separately entering the regression model, the coefficients of E2 changed to –0.052→-0.052→-0.058. When Hcy entering the regression model, the coefficients of CGRP changed from -0.022 to -0.018, the coefficients changed little (Table 7). This showed the three factors are independent.

**Table 6:** Estimated parameters for Logistic regression model for CHD

Variables		Coefficient	SE	P Value	Odds Ratio	95% CI for Coefficient	
						Lower	Upper

Step1	E_2_	-0.052	0.012	<0.001	0.949	0.927	0.973
	Constant	4.667	1.220	<0.001	106.33		
Step2	E_2_	-0.052	0.014	<0.001	0.949	0.923	0.976
	CGRP	-0.022	0.007	<0.001	0.978	0.965	0.991
	Constant	70720	1.841	<0.001	2253.6		
Step3	Hcy	0.264	0.103	<0.001	1.302	1.065	1.593
	E_2_	-0.058	0.106	<0.001	0.944	0.915	0.974
	CGRP	-0.018	0.007	<0.001	0.982	0.969	0.995
	Constant	4.671	1.943	<0.001	106.83		

**Table 7:** Confounding influence on logistic regression: Regression coefficients of a sequential analysis

Constant	E_2_	CGRP	Hcy

4.667			
	-0.052		
	-0.052	-0.022	
4.671	-0.058	-0.018	0.264

## DISCUSSIONS

Numerous possible risk factors for coronary heart disease (CHD) have been identified. In the present study, we found that old ages, diabetes TG and hypertension are all independent risk factors for CHD in postmenopausal women, which is consistent with prior reports (18). Hcy is a sulfur-containing amino acid, formed from methionine as a product of numerous S-adenosylmethionine-dependent transmethylation reactions. Two enzymes utilize Hcy, including tathionineβ-synthase(CBS) and methionine synthase(MS)(19). A very wide range of effects has been attributed to Hcy. These include direct damage to endothelial cells, flawed platelet activity, elevated procoagulant activity, increased collagen synthesis, and enhanced proliferation of smooth muscle cells. Biochemically, it has been proposed that Hcy modifies eicosanoid metabolism, promotes translocation of protein kinase C from cell nuclei and cyto-plasm to the cell membranes, and induces c-fos and c-myb activity (20-24). In this study we found that an elevated increase in Hcy level is associated positively with higher scores for severity of coronary atherosclerosis, which is also consistent with other case control studies (1-5). Hcy levels increase with age in women, but the mechanism is not clear (25-26). In the present study, the mean serum Hcy level was significantly higher in CHD postmenopausal women than in NCHD postmenopausal women and young subjects. However, a multivariate logistic regression analysis showed no relationship between Hcy and E2 levels.

CGRP is a sensory neuropeptide with potent vasodilatory and cardiotonic actions. It is a more potent vasodilator than other endogenous or synthetic compounds, which include prostacyclin, adrenaline, histamine, vasoactive intestinal polypeptide, and sodium nitroprusside. Furthermore, CGRP is present in the adventitial nerves surrounding most peripheral blood vessels, including coronary and cerebral vessels (6-7). In the present study, the mean serum CGRP level is significantly lower in CHD postmenopausal women than in NCHD postmenopausal women. In the multivariate logistic regression analysis, CGRP was an independent protective factor in the development of coronary arteriopathy in women, but there was no relationship between CGRP and E2 level.

In recent years people have begun to pay much more attention to the relationship between CHD and hormone replacement therapy, as well as health benefits and risks of hormone therapy. HERS II study indicated that after 6.8 years, hormone therapy did not reduce risk of cardiovascular events in women with CHD. Results from WHI study showed that overall health risks exceeded benefits from the use of combined estrogen and progestin for an average 5.2-year follow-up among healthy post-menopausal US women (27-28). In the present study, we found that there is no relationship between CGRP and endogenous female gonad hormone (E2) in postmenopausal women without hormone therapy. That is, endogenous female gonad hormone (E2) neither increases serum CGRP levels (which is a protective factor to CHD) nor drops serum Hcy levels (which is a risk factor to CHD) in postmenopausal women with CHD. However, the fact is that postmenopausal women do have higher rates of CHD than young women, of which there is no simple explanation. The decline of endogenous female gonad hormone levels with age is accompanied by other pathophysiologic changes, such serum lipids, etc. The relationship between female gonad hormone and CHD will continue to remain an important topic.

Furthermore, serum lipids are risk factors for CHD. In the present study, we found no differences in serum lipid levels (TCHO, HDL-C, LDL-C) between the CHD and NCHD groups. This may be due to the limits in choosing patients from the patient population. All of the patients that underwent diagnostic coronary angiography have been treated for the secondary prevention of cardiovascular disease (including lipid-therapy).

## CONCLUSION

The results of the present study show that Hcy is an independent risk factor in the development of arteriopathy in postmenopausal women. CGRP and endogenous E2 are independent protective factors in the development of coronary arteriopathy. There are no relationships between Hcy, CGRP and endogenous E2 levels.
